# Wearability Assessment of a Wearable System for Parkinson's Disease Remote Monitoring Based on a Body Area Network of Sensors

**DOI:** 10.3390/s140917235

**Published:** 2014-09-16

**Authors:** Jorge Cancela, Matteo Pastorino, Alexandros T. Tzallas, Markos G. Tsipouras, Giorgios Rigas, Maria T. Arredondo, Dimitrios I. Fotiadis

**Affiliations:** 1 Life Supporting Technologies, Universidad Politécnica de Madrid, Avenida Complutense 30, Madrid 28040, Spain; E-Mails: jcancela@lst.tfo.upm.es (J.C.); mpastorino@lst.tfo.upm.es (M.P.); 2 Unit of Medical Technology and Intelligent Information Systems, Dept. of Materials Science and Engineering, University of Ioannina, 45110 Ioannina, Greece; E-Mails: atzallas@cc.uoi.gr (A.T.T.); markos@cs.uoi.gr (M.G.T.); rigas@cs.uoi.gr (G.R.); fotiadis@cc.uoi.gr (D.I.F.)

**Keywords:** compliance, Parkinson's disease, Body Area Network (BAN), remote monitoring, motor assessment

## Abstract

Wearable technologies for health monitoring have become a reality in the last few years. So far, most research studies have focused on assessments of the technical performance of these systems, as well as the validation of the clinical outcomes. Nevertheless, the success in the acceptance of these solutions depends not only on the technical and clinical effectiveness, but on the final user acceptance. In this work the compliance of a telehealth system for the remote monitoring of Parkinson's disease (PD) patients is presented with testing in 32 PD patients. This system, called PERFORM, is based on a Body Area Network (BAN) of sensors which has already been validated both from the technical and clinical point for view. Diverse methodologies (REBA, Borg and CRS scales in combination with a body map) are employed to study the comfort, biomechanical and physiological effects of the system. The test results allow us to conclude that the acceptance of this system is satisfactory with all the levels of effect on each component scoring in the lowest ranges. This study also provided useful insights and guidelines to lead to redesign of the system to improve patient compliance.

## 1. Introduction

During the last decade, we have witnessed an incredible advancement in the growth of wearable technologies for health monitoring and long-term monitoring of patients and healthy subjects, in their daily lives. Nowadays, health monitoring applications often integrate different sensors into a sensor network around the subject or the patient. In the case of the PERFORM system a Body Area Network (BAN) was designed to monitor all major symptoms of Parkinson's disease (PD).

The use of these systems can be highly intrusive and uncomfortable for the patients and this could lead the application to fail, even when it has been proven that it benefits patients' health. Therefore, compliance must be taken into account during the development of any wearable system in order to identify potential weak points in the adoption of the systems by the final users. Nevertheless, during the prototyping phase most works focus exclusively on the technical performance of the system and ignore or bypass the wearability and compliance analysis. This work presents a wearability assessment which complements the papers [1–4] that we have already published describing the technical performance for the detection and quantification of PD system using a BAN of sensors. Our analysis might be extended to other similar systems, since most of factors have been taken into account.

### 1.1. Parkinson's Disease

According to the World Health Organization (WHO), neurodegenerative diseases are considered to be among the most burdensome [5]. PD is one of the most common neurodegenerative disorders. It occurs in about 1% of the population over the age of 60 and its prevalence increases with age [6]. Advancements in treatment for chronic diseases have resulted in reduced length of hospital stay, and in some cases, the avoidance of hospital visits. Telemedicine brings healthcare delivery to the home environment by connecting the patient with medical professionals. It is not intended to replace health professional care, but rather to enhance the level of care [7]. The most remarkable motor disorders in PD are resting tremor, rigidity, bradykinesia (*i.e.*, slowness movement), hypokinesia (reduced amplitude movements) and postural instability [8]. These motor symptoms are strongly linked with the loss of dopaminergic innervation of a region named basal ganglia [9]. The origin of this loss has been largely studied and several risk factors for PD have been identified [7,10,11] as well as a genetic predisposition. The cause and etiology of PD are largely unknown [10–14]. As the disease progresses, PD patient's motion becomes slower and tremoric and the response to medication fluctuates along the day (ON-OFF periods). In addition, the presence of involuntary movements (dyskinesias) deteriorates voluntary movement in advanced stages of the disease.

### 1.2. The PERFORM System

The PERFORM System is a wearable platform for PD remote monitoring and management [9,15]. Currently a fully operative prototype has been tested in three European hospitals: Clínica Universidad de Navarra (Spain), the University of Ioannina Hospital (Greece) and the Nuovo Ospedale Civile S.Agostino-Estense of Modena (Italy). Nowadays, motor assessment in PD is mainly based on historical information, home diaries and neurological examination during visits to the clinic. These methods clearly suffer from many drawbacks: data from these sources can be highly subjective, they rely on the patient's memory and perception of his own symptoms and they depend on the physician's experience. Moreover, most of the patients may not be aware of mild symptoms, they may not necessarily understand medical terminology, or they may unconsciously exaggerate or attenuate symptom severity. The development of this system is aimed at providing patients and clinicians with a tool for the continuous and objective monitoring of PD symptoms.

**Figure 1.**
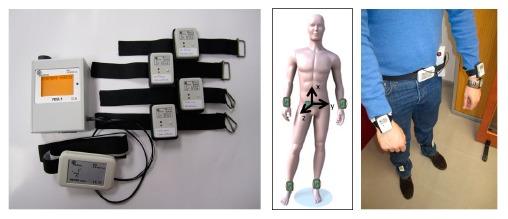
Sensors used for data collection and their positioning in the patient body [9,15,16].

The platform is composed of a set of wearable sensors for the recording of the motion signals and algorithms for the processing of physiological signals (Figure 1). The hardware consists of a set of four tri-axial accelerometers positioned on each limb of the patient used to record signals from legs and arms; a belt sensor, composed of an accelerometer and a gyroscope, used to record body movement accelerations and angular velocity; and a data logger used to receive and store all recorded signals in a SD card. All sensors send data using the Zigbee protocol to the logger device, which has a 62.5 Hz sampling rate before a synchronization phase. All accelerometers transmit data at the same time and no retransmission of lost packets has been implemented to save energy.

For each symptom, an *ad hoc* algorithm processes the relevant signals, detects the symptom episode and quantifies it on a severity scale from 0 to 4, according to the UPDRS scale for PD patients [17]. The system shows an accuracy 93.73% for the classification of levodopa-induced dyskinesia (LID) severity [1], 86% for bradykinesia severity [2] and 87% for tremors [3]. A special module for the assessment of the gait parameters *i.e.* step frequency, velocity, arm swing frequency and entropy of the gait signal (entropy is a good measure of the randomness of the signal and it is an excellent discriminator of the On/Off status) has also been developed [4]. These results were obtained using various classifiers (e.g., support vector machines, decision trees, random forests, *etc.*). Once the technical performance was assessed the wearability assessment described in this work was carried out in order to evaluate the acceptance of the system by the final users and to identify areas of improvement in terms of design. Within the project an iterative design process was followed. Three different phases were designed involving 92 PD patients and 20 healthy subjects. Each phase of the design raw signals were collected and processed:

- Phase I. Preliminary test of the technical performance with healthy subjects.
- Phase II. To validate not only the technical performance of the system but also the clinical compliance of the system.
- Phase IIII: It consisted of the evaluation of final prototype at patients' home.

In this work a methodological approach has been followed in order to extract and analyze the PD patients' feedback regarding the compliance of the BAN system. To carry out this compliance assessment different already validated methodologies have been emplaced, specifically the REBA, Borg and CRS scales have been used in combination with a body map when needed. A total of 32 PD patients have been involved in this study, showing an excellent acceptance of the BAN system in wearability terms. The overall result of the evaluation can be broken down into the subsequent marks: average perceived exertion score (0.71 on the Borg CR-10 scale), average posture score (0 based on the REBA scale), average localized pain/discomfort score (0.11 on the Borg CR-10 scale), average emotion score (1.4 on the CRS scale), average attachment score (2.5 on the CRS scale), average harm score (0.15 on the CRS scale), average perceived change score (0.67 on the CRS scale), average movement score (0.59 on the CRS scale) and average anxiety score (1.63 on the CRS scale). Each individual component of the wearability assessment can be labeled as a low level effect according to the guidelines provided for each of the methodologies.

## 2. Experimental Section

### 2.1. Participants

Subjects fulfilling the following criteria were eligible for the study: diagnosis of PD, aged between 40 and 70 years old, ambulatory, capable of complying with study requirements, experiencing motor fluctuations, receiving stable dopaminergic treatment and with the support of a caregiver who can cooperate with the patient and the doctor. Participants suffering from dementia, hallucinations or any significant systemic disease were excluded from our study.

**Figure 2.**
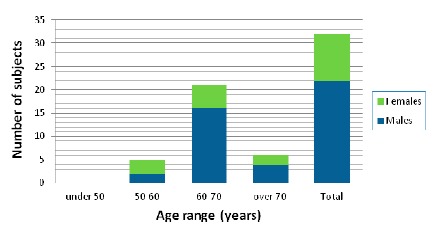
Age distribution of participants in our study.

Before the enrollment of the patients in the study, they were provided with a detailed document describing the study in plain language as well as an oral explanation of the research expressed in terms that would have the best chance of being understood. The experimental nature of this study, its inherent risks and drawbacks, and its chance of improving the treatment of PD were discussed. Then the clinician obtained informed consent and gave a letter with a synopsis of the study protocol for the family doctor to inform him about main issues of the study. A total of 32 participants with the age and gender distribution shown in Figure 2 were involved in our study.

### 2.2. Wearability Assessment

Wearable systems differ from portable technologies, such as mobile phones, which are designed to be carried. Even mobile phones, “worn” in a holster attached to a belt, are often detached and held in the hand when dialling or texting, and as such are merely being carried until they are used [18]. For wearable computer systems, the term “wearability” has been defined as the use of the human body as a support system for some product [19].

Wearability directly impacts the usability of a wearable computer system. If the wearer is uncomfortable or placed under too much stress then the usability of the system will be reduced. Also, it can highlight areas for concern and indicate needs for improvement in the design of the product [18]. Usability is not simply a product of the device but a consequence of the interaction between the person and the device [20].

The wearability evaluation of a device is a multifaceted problem; wearable devices affect the wearer in different ways and thus, there are several effects that should be taken into account when assessing the wearability of the device. These effects range from physiological, due to attachment of an extra load to the human body, to comfort related effects. Furthermore, there exist a number of methods for measuring physiological and biomechanical variables that could be employed for the evaluation of wearable computers. However, these methods usually employ expensive equipment which requires technical expertise and are often limited in their applicability in real world settings due to the size of the equipment, lack of portability and need for calibration. These methods are therefore preferred for lab-based experiments, rather than user trials and specially trials in patients' homes. For this kind of trials, noninvasive and non-restrictive methods are required, which are relatively quick and easy to implement, and require minimal interference with the user during data acquisition. Therefore, observation and questionnaire-based methods are preferred. The wearability evaluation of BAN is mostly based on the work of Knight and Baber [18]. Attaching a load to the body, as it does any wearable computer equipment, can have a number of physical effects that should be assessed to ascertain its wearability. These effects include [18]:

- Physiological—This refers to the level of energy spent when performing a specific activity, which may include perceptions of exertion and physical fatigue. An attached load to the body will have a direct effect on the energy expended by the body, as the muscles burn more energy to generate force to counteract the weight of the load. In addition, energy expenditure may also increase due to movement and posture alteration [18]. For measuring the physiological effects which monitor the heart rate is perhaps the most common method of estimating energy expenditure. Since none of the parameters are measured by the available sensors, it was decided not to be used as an extra sensor would be needed. The method selected is the BORG CR-10 scales for perceived exertion [18] usually employed to measure the perceived exertion while exercising [21]. In this study the user will need to point out the extra exertion the patient has given due to the fact that he is wearing the wearable, while carrying out their daily activities.
- Biomechanical—This refers to musculoskeletal loading, which may be localised and may result in perceptions of muscle fatigue. It also refers to changes in movement patterns and posture. Knight and Baber recommended assessing the postures adopted when interacting with the wearable device to determine the level of postural loading [18] and also the effect of adding load to the body in the form of a wearable device.
- Since in this case the wearers must wear the device while performing daily activities, this study did not intend to emphasize on specific postures but rather making sure that the device does not obstruct the users in any of the normal postures. Our study of the biomechanical aspects has been derived into two activities:
  ○ Posture and movement, which have been assessed using the Rapid Entire Body Assessment (REBA) methodology [22].
  ○ Musculoskeletal loading need to be undertaken, regarding to the weight of the wearable. Measurements of loading can be made by employing electromyography to measure levels of muscular exertion. However, these methods are best applied in controlled experimental conditions, rather than user trials. An alternative method is to use rating scales, such as the Borg CR-10 used in conjunction with body maps [21].
- Comfort—This refers to localised discomfort due to musculoskeletal loading, as well as a general sense of well-being. It may also include discomfort due to stresses placed on specific physiological systems (e.g., head mounted displays and the visual system). The comfort assessment of the device needs to take into account several dimensions. Knight and Baber developed a tool for the comfort assessment of wearable devices called the CRS [23] which break down the comfort analysis into six different components.

**Figure 3.**
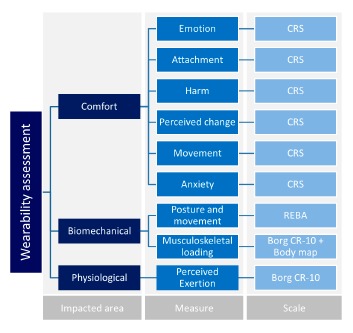
Wearability assessment scheme. CRS (Comfort Rating Scales), REBA (Rapid Entire Body Assessment).

Assessing the wearability of a given wearable systems could thus involve an evaluation of the physiological, biomechanical and comfort effects. For each category of effects, the selected methods and scales are shown in Figure 3 [18].

So far, a set of measures and tools has been proposed but the determination of this evaluation requires the convergence of the measures of the three axes (comfort, biomechanical and physiological) and their nine branches into one wearability score. To determine such a score several criteria have been developed using the methods discussed above [18]. These criteria are summarized in Table 1. For each measure, the value obtained from an assessment can be associated to a level of effect ranging from “Low” to “Extreme”. These levels were determined from values published for the descriptors used on the CR-10 scales [21], and the action levels published in the REBA method [22]. For the comfort scales the levels of effect were determined by proportioning the scales into equal parts [18,23].

From the levels of effect, five Wearability Levels (WL) have been proposed by Knight and Baber, as shown in Table 2 [18]. They allow the homogenous interpretation of the different metrics and scales in an easy and direct way providing a final score rating the wearability of the system.

#### Tools for Wearability Assessment

The aim of this section is the description of each of the scales mentioned above, and the explanation of how they can be used in the context of the wearability assessment of the system.

**Figure 4.**
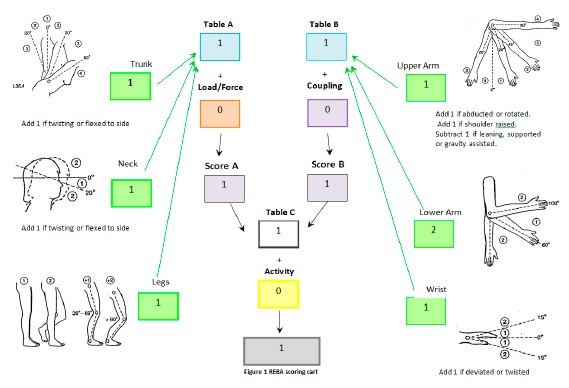
Example of the REBA scoring card used during PEFORM pilots based on the Hignett and McAtamney scheme [22].

**Figure 5.**
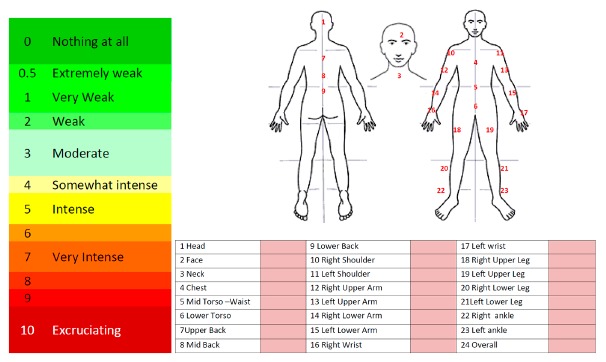
Borg CR-10 Scale and body map.

(a) Rapid Entire Body Assessment (REBA)
  Changes in posture and movement can result in perceptions of physiological exertion, muscle exertion and discomfort. Over a short time these can develop into perceptions of fatigue and pain, and in the long term they have been associated with chronic musculoskeletal problems. Posture and movement can be assessed using opto-electronic devices [18]. However, these methods are often expensive and time consuming. Alternatively, posture and movement can be assessed using observational methods. An example of these methods is the REBA method developed by Hignett and McAtamney [22]. The REBA method has been developed to fill a perceived need for a practitioner's field tool, specifically designed to be sensitive to the type of unpredictable working postures found in healthcare and other service industries [22].
  Most postural analysis techniques have two complementary qualities, generality and sensitivity [24]. High generality in a postural analysis method may be compensated by low sensitivity [22]. A need was perceived within the spectrum of postural analysis tools, specifically with sensitivity to the type of unpredictable working postures found in healthcare. This lead to the development of the Rapid Entire Body Assessment (REBA) tool [22] which aims to develop a postural analysis system sensitive to musculoskeletal risks for a variety of tasks and only require a minimal equipment (a paper and a pencil).
  For each body region, there is a posture scoring scale and additional adjustments which need to be considered and accounted in the score. To carry out the REBA assessment the evaluator is provided with a scoring card showing some schemes to help the evaluator to understand some of the questions [22]. Figure 4 shows an example of the evaluation performed during the assessment. The steps we employed to carry out the REBA assessment for the system are:
  - Step 1: Perform the neck, trunk and leg analysis.
  - Step 2: Calculate the score for Load/Force.
  - Step 3: Using values from steps 1 and 2, locate the, so called, Score A.
  - Step 4: Perform the upper arm, lower arm and wrist.
  - Step 5: Calculate the score for Coupling.
  - Step 6: Using values from steps 4 and 5, locate the, so called, Score B.
  - Step 7: Using values from steps 3 (Score A) and 6 (Score B), locate the REBA.
(b) Borg Scales
  The Borg Relative Perceived Exertion (RPE) scale is used to measure the overall physiological effort and it is a common method for determining exercise intensity levels [18]. The RPE scale measures, in a quantitative way, the work that the user needs to put to wear the device. The generally used scale is based on the research carried out by Gunnar Borg [25]. There are different versions of this scale, the two best well-known are:
  - Borg 6–20—The original scale as developed by Borg, ranging from 6–20 (which is related to 1/10 of the exercise heart rate).
  - Borg CR-10—An updated scale by Borg with the ratings ranging between 0 (nothing at all) and 10 (very hard) [21].
  The CR-10 scale is specially indicated to identify a preponderant sensation coming from a specific area of the body, for example, muscle pain. In these cases, the scale is used together with a body map, in this way the user can self-assess the perceived exertion on each body part (Figure 5).
(c) Comfort Rating Scales (CRSs)
  When wearing something, the level of comfort can be affected by a number of parameters, such as the device's size and weight, how it affects movement, and pain, whether direct (e.g., friction, knocking, heat) or indirect (e.g., muscle fatigue) [23]. In addition to physical factors, comfort may be affected by psychological responses such as embarrassment, especially in the case of medical devices where wearing a device could disclosure that the subject is suffering a certain pathology. Consequently, Knight and Baber proposed that comfort should be measured across a number of dimensions and for such task they developed the Comfort Rating Scales (CRSs) [23].
  The CRSs have been developed to provide a quick and easy-to-use tool to assess the comfort of wearable computers. Using the scales may assist researchers and designers of wearable computers in measuring total wearer comfort and highlight factors that should be addressed to improve comfort [23]. The CRSs attempt to gain a comprehensive assessment of the comfort status of the wearer of any item of technology by measuring comfort across the six dimensions:
  - Emotion—Concerns about appearance and relaxation.
  - Attachment—Comfort related to non-harmful physical sensation of the device on the body (e.g. the feel of the device either directly as pressing on the body or indirectly as it pulls on clothing or moves in relation to the body).
  - Harm—Physical sensation conveying pain.
  - Perceived change—Non-harmful indirect physical sensation making the wearer feel different overall with perceptions such as being awkward or uncoordinated, may result in making conscious compensations to movement or actions.
  - Movement—Conscious awareness of modification to posture or movement due to direct impedance or inhibition by the device.
  - Anxiety—Worries as to the safety of wearing the device and concerns as to whether the wearer is using it correctly or it is working appropriately.
  The CRSs are 21-point scales anchored at each end with the labels low and high. A 21-point scale was used so that scoring on the scale would range from 0 at the far left to 20 at the far right [23]. According to Knight and Baber this range was considered large enough to elicit a range of responses that could be used for detailed analysis. In rating perceptions of comfort, the scorer simply marks on the scale his or her level of agreement, from low to high, with the statements made in the “description” column of Table 3. These statements were devised based on the interpretation of the aspect of comfort each group conveyed.

### 2.3. Data Collection

Based on the above methods of assessment, a wearability questionnaire was elaborated to be used during wearability evaluation and it is composed of six sections (Table 4).

It is noted that each section is preceded by short guidelines for the interviewer. The evaluation procedure starts with the Posture Analysis, based on REBA evaluation.

- All patients were asked to stand up and stay still for a couple of minutes, while an observer completed the REBA evaluation scoring card based on the posture of trunk, neck, legs and arms. Photographs were also taken to capture their current posture (prior to wearing the system).
- Then the patients were asked to wear the prototype device and the same procedure was repeated. This second posture evaluation had similar results for every single body part of each patient respectively; obviously resulting to similar REBA scores.
- As it was previously mentioned, the REBA evaluation in PD patients is meaningful if the observer differentiates between the postural changes caused by the disease and any postural changes caused by the wearable itself. Thus, in order to identify these changes we use as score for our evaluation the difference between the REBA scores the patient gained (i) without and (ii) with the wearable device. This helps us to focus the posture analysis on the effects imposed by the wearable itself.

In the second step of the evaluation process the patients were asked to make a self-assessment of their own perceived energy cost due to the load of the wearable device, using the Borg CR-10 scale for energy cost.

In the third step of the evaluation process the patients were asked to provide a rating from 0 to 10 representing their own feeling of pain/discomfort for each body part (Borg CR-10 scale for pain/discomfort).

Finally, in the last section of the evaluation the patients were asked to read a number of statements and to provide a rating between Low (0) and High (20) corresponding to their level of agreement with each statement. The patients' responses are indicated in the following sections.

## 3. Results and Discussion

For better comprehension of the results all the perceived exertion and emotion were translated into a homogenous five-level scale following the criteria of Table 1.

### 3.1. Physiological

As it was already mentioned a wearable device is an extra load that gets attached to the human body. Thus, it could have a direct effect both on the energy consumed by the body as the muscles need more energy in order to produce the force to counteract the weight of the device and the resulting movement and posture changes. The physiological effects study focused on the analysis of the self-assessment perceived exertion by the patients.

#### Perceived Exertion

**Figure 6.**
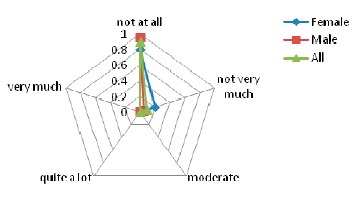
Results for the perceived exertion evaluation.

As indicated in Figure 6, the majority of patients did not feel any exertion caused by the device. A moderate feeling of physical stress was reported by only one 71 year old female patient.

### 3.2. Biomechanical

Apart from the extra energy needed in order the muscles to perform the necessary movements, the device can cause musculoskeletal loading. In some cases, the loading might be localised and result to perceptions of muscle fatigue. In this case the results presented here are the posture and movement analysis based on the REBA score with and without wearing the device and the musculoskeletal loading based on the self-assessment of the patients with a Borg CR-10 scale and a body map.

#### 3.2.1. Posture and Movement

**Figure 7.**
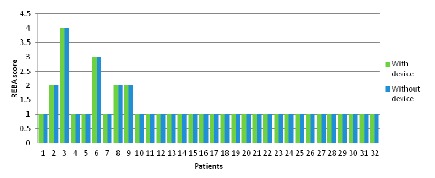
Posture analysis results using REBA scale.

As indicated in Figure 7, the interpretation of this outcome is that the wearable device did not have any impact on the patients' posture.

#### 3.2.2. Musculoskeletal Loading

The responses reported in Table 5 reveal that the majority of the patients did not feel any discomfort/pain on almost any parts of their body. Only a few patients (eight out of 32 patients) felt extremely weak discomfort/pain at the overall assessment of their body and at some parts of their body such as: Mid Torso-Waist, Mid and Lower Back, Right and Left Wrist, Right and Left ankle. According to Level of effect described in Table 1, all the affected areas are coded as “Low”. The most remarkable effects in the Mid Torso-Waist, Mid Back and Lower Back are a direct consequence of the data logger impact (the heaviest and largest device of the BAN).

### 3.3. Comfort

Apart from muscle fatigue, the musculoskeletal loading might could also lead to discomfort or affect the general sense of well-being. It may also lead to discomfort due to stresses placed on specific physiological systems. All the results are expressed as perceptual values ranging from 0 to 1 and grouped according to Table 1.

#### 3.3.1. Emotion

**Figure 8.**
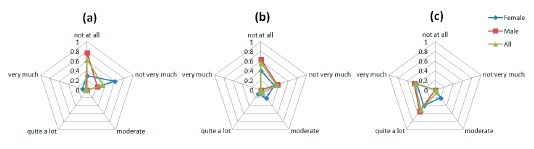
Comfort results regarding the emotion factor. The questions asked to the users were: (**a**) “I feel worried and embarrassed”; (**b**) “I feel tense” and (**c**) “I would wear the device if it was invisible”.

The graphs in Figure 8 are indicative of the patients' responses on the emotion-related questions of the Comfort Rating Scale questionnaire. The social impact from wearing the sensors and being seen by other people is a sensible concern for the PD patients involved in the study. The majority of the patients did not feel very very tense wearing the device. However, most of the participants agree that they would not mind wearing the device if it was invisible as depicted in the Figure 8c. As it was mentioned above, part of this concern could come from the public disclosure of suffering a disease only by wearing a device.

#### 3.3.2. Attachment

**Figure 9.**
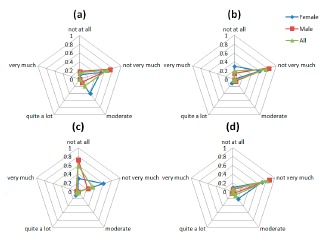
Comfort results regarding the attachment factors, the questions asked to the users were: (**a**) “I feel the device on the body”; (**b**) “I feel the device moving”; (**c**) “I was not able to move as usual” and (**d**) “I have difficult in putting on the device”.

The graphs in Figure 9 indicate that patients have some difficulties related to the attachment of the device to their body. Some of them tried to be careful with their movements out of fear of dropping or disconnecting any sensor, also the attachment method, and the requirement of tighten the straps to ensure a good technical performance of the system, has a moderate impact in the comfort of the patients (Figure 9a). Also, the straps methods led to some difficult to wear the device without a care giver help. Finally, the answer to the (Figure 9c) question shows that the system does not restrict the daily movement and activities of the patients.

#### 3.3.3. Harm

**Figure 10.**
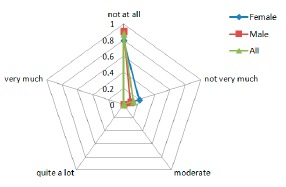
Comfort results regarding the harm factor, the question asked to the users was: “The attached device causes me some kind of harm”.

Results regarding the Harm (indicative of the subjects' perception of any kind of harm e.g., headache, pain, itching, irritation, *etc.*) caused by the device are shown in the Figure 10. The feedback received is that none of the patients had such a concern, ranging in their answers from Not at all and Not very much.

#### 3.3.4. Perceived Change

**Figure 11.**
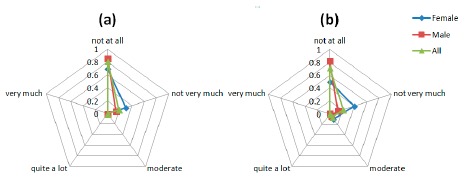
Comfort results regarding the perceived change factors, the questions asked to the users were: (**a**) “I feel more bulky” and (**b**) “I feel change in the way people look at me”.

Figure 11 shows the results for the Perceived change questions. Again the social component appears again since some patient expressed their concerns about what other people could think about them (Figure 11b).

#### 3.3.5. Movement

**Figure 12.**
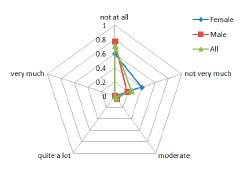
Comfort results regarding the movement factor, the question asked to the users was: “The device obstructs my movements”.

As far as the impedance of movements is concerned, the received feedback is depicted in Figure 12. In general, all participants in the study agree that the system did not obstruct or block them in everyday activities neither limit their activities.

#### 3.3.6. Anxiety

**Figure 13.**
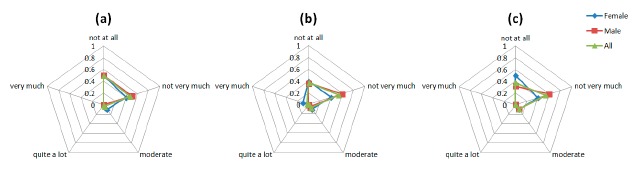
Anxiety results regarding the perceived change factors, the questions asked to the users were: (**a**) “I do not feel secure with the device”, (**b**) “I feel that I do not have the device properly attached” and (**c**) “I feel that the device is not working properly”.

A constant moderate concerning is shown in all the questions regarding the Anxiety space (Figure 13). The anxiety caused to patients by the device is generated by a lack of feedback to patient about the correct placement and functioning of the device.

### 3.4. Interview and Complementary Questions

The last section of the wearability study was an informal interview with the patients where they were allowed to freely express their concerns and feelings about the device. The meeting with the users raised some appearance and emotional issues among a substantial group of patients (six out of 32). This subset of patients had some concerns about others' opinions when wearing the device. They would feel much more comfortable wearing an invisible device or if the wearing time were limited to the home environment. Apart from appearance aspects, they experienced no particular problem with the device.

As far as the rest of the patients are concerned, most of them had absolutely no substantial problem with the device. In fact they have forgotten of wearing it after a while. There were also patients who wouldn't mind wearing it both in and outside their home. We hereby present some statements as given by patients:

- “The device created a little bit of stress and nervousness”
- “I felt weird due to the presence of other persons in the same space”
- “I felt embarrassment because I thought that everybody was looking at me”
- “I encountered some problems to wear the device at home”
- “My only concern is that I may encounter some difficulties in attaching properly the device if I am in OFF state; the same problem I have also for dressing up.”
- “I experienced no problem at all while wearing the device.”
- “After some rehearsals I will be able to attach the device myself without help.”

As far as the ability to wear and attach the device properly on their own, most patients replied that they would need some help from their caregivers. On the other hand, the device would not prevent them from doing their daily tasks.

Also, a common concern raised during the interview was if the devices were waterproof since some daily and very common activities could wet the devices, especially the ones on the wrists, for example when washing the hands and washing dishes. Afterwards all patients responded to a number of questions related to allergic reactions, excess heat and sweat caused by the device. Table 6 summarizes the number of positive and negative responses to the allergic reaction received by the total number of patients. As it is shown no problem was encountered by any of the participants.

### 3.5. Overall Evaluation

The overall level of effect of the device on patients is summarized in Table 7. To calculate the average CRS score of the e1.3 value in the emotion section the answers of the patients were transformed in to mirror image over the CRS scale, e.g., an answer of 19 was transformed to 1 and vice-versa. This was needed to make the e1.3 homogeneous with all the other comfort values, where the lowest the value the better in terms of wearability. After this change, it was possible to calculate the average value of the emotion section.

## 4. Conclusions

In this work we have performed a wearability analysis of a particular system, which is used for the monitoring of patients with Parkinson's disease. The system is based on a set of sensors and analysis of the acquired signals using intelligent algorithms. The analysis performed can be extended to the wearability analysis of similar systems for movement disorders monitoring or other diseases which require the use of sensors for the continuous monitoring of physiological parameters. The proposed methodology is not applicable when implanted or invasive sensors are used, since other parameters must be taken into account. The approach can be based on a similar methodology, but it differs since other sensors used present different features that must be evaluated, such as weight, battery operation, placement, *etc.*

The evaluation of the system will be benefited from extending the analysis to a larger number of patients. To our knowledge the number of patients used in our study is large enough compared to other wearability studies [26–29] and evaluation of monitoring systems for the Parkinson's disease and other movement disorder diseases.

The results of the wearability assessment are summarized in Table 7 and indicate that the general level of effect of the system is “Low” since all scores are “Low” for each category of assessment. Nevertheless, analyzing the charts results in detail, it is possible to extract some weak points and areas of improvement for the system under assessment.

Starting this analysis with the energy cost and the biomechanical spaces the low scores for the perceived exertion and the posture values suggest that even the prototype is larger and heavier than a commercial device it does suppose a substantial physical load for the users. The results of the localized pain/discomfort confirm this suggestion. The moderate scores in the mid torso-waist, mid back and lower back lead to identify the weight of the data logger device as the part of the BAN more penalized by the users in terms of physical impact and energy cost. A direct action to palliate this is to reduce the weight and size of the data logger device. Another option could be to modify the attachment method. Using a strap as attachment of the data logger device to the subject makes that all the force generated by the logger weight is applied in the subject's waist. Transforming the data logger into portable device to wear in the pocket could improve this and, at the same time, and thanks to the advances in the smartphone devices, currently the data logger device could be integrated into a commercial smartphone.

Regarding the comfort analysis, both the emotion and anxiety results suggest a significant concern of the patients about their privacy, most of the patients do not like what other people could think about them when they wear the device, especially in public spaces. Feelings of anxiety indicate levels of discomfort arising from concerns about safety and reliability and from feelings of insecurity [23]. One method to reduce this apprehension could be to make the device less noticeable by reducing the size of the device, hiding it within clothing or camouflaging it as an everyday item of clothing. Ankle sensors could be hidden under the clothes and the wrist sensors could be modified to imitate bracelets. Regarding the logger device, again, a good option could be to substitute it by a portable device that could be carried in a pocket. Ultimately, levels of both emotion and anxiety may be reduced with time and experience as the wearer becomes accustomed to the device [23].

About the attachment which according to the users is the weakest point of the system, it has been confirmed with the informal interviews that the use of the straps lead to some unpleasant feelings in terms of comfort but also, it makes very difficult to users the self-wearing of the device, especially the wrist sensors. The way that patients wear and attach the devices or sensors properly on their own is difficult and uncomfortable and they might need some help from their caregivers.

Likewise, the moderate impact of the perceived change results suggests that the users expect to receive more feedback from the system about the correct functioning or at least a procedure to know if the system is working properly. In general a better understanding of the device and how it works could address the anxiety of the patients, therefore providing better training material in a friendly way could help to reduce this concern, but the system should also be improved to provide an easy to understand feedback to the patients at any time, and in the case some error is risen a clear message must be provided with the proper instructions to overcome or to report such a problem.

Finally, it is also important to ensure waterproofing of the sensors, or at least for the wrist sensors. Although, this should not be a restrictive requirement, since the goal is not to dip the sensors, for example to take a bath, but to allow the user to carry out daily activities such as washing the hands and washing the dishes, activities where the sensors could eventually get wet.

To summarize, the data logger device should be transformed into a portable device or even integrated into a currently available smartphone platform. The wrists sensor should be evolved to make it more imperceptible either by reducing the size or by imitating some item of clothing and waterproof. Also, the attachment method should be enhanced to facilitate the self-wearing of the device. Finally, the ankle sensors have not received any special critique but the attachment method could be also improved. And, in any case, it seems important to design sensor devices or sensors that could not be easily spotted by other persons.

## Figures and Tables

**Table 1.** Level of effect of the different scales for the wearability of a system [18].

			**Level of Effect**				
	**Metric**	**Units**	**Low**	**Moderate**	**Large**	**Very Large**	**Extreme**
Energy cost	Relative perceived exertion	Borg CR-10 score	0–1	2–3	4–5	6–7	8–10
Biomechanical	Posture	REBA score + body map	1	2–3	4–7	8–10	11–15
	Localised pain and discomfort	Borg CR-10 score	0–1	2–3	4–5	6–7	8–10
Comfort	General wearable	CRS score	0–4	5–8	9–12	13–16	17–20

**Table 2.** Level of effects and corresponding wearability levels [18].

**Level of Effect**	**Wearability Level**	**Outcome**
Low	WL1	System is wearable
Moderate	WL2	System is wearable, but changes may be necessary, further investigation is needed
Large	WL3	System is wearable, but changes are advised. Uncomfortable
Very Large	WL4	System is not wearable, fatiguing, very uncomfortable
Extreme	WPL5	System is not wearable, extremely stressful and potential harmful

**Table 3.** Questions for the CRSs evaluation.

**Question Code**	**Description**
e1.1	I feel worried and embarrassed.
e1.2	I feel tense.
e1.3	I would wear the device if it was invisible.
a2.1	I feel the device on the body.
a2.2	I feel the device moving.
a2.3	I was not able to move as usual.
a2.4	I have difficult in putting on the device.
h3.1	The attached device causes me some kind of harm.
pc4.1	I feel more bulky.
pc4.2	I feel change in the way people look at me.
m5.1	The device obstructs my movements.
an6.1	I do not feel secure with the device.
an6.2	I feel that I do not have the device properly attached.
an6.3	I feel that the device is not working properly.

**Table 4.** The main sections of the wearability analysis.

**Section**	**Name**	**Description**
1	Personal information	Age Sex
2	Posture Analysis	To be completed by an observer & includes all the material related to the REBA score chart
3	Energy Cost Analysis	To be completed by the patient according to his perception of extra energy used in order to perform daily activities
4	Pain/Discomfort Analysis	To be completed by the patient according to his perception of pain or discomfort to specific body areas (using the body maps)
5	Pain/Discomfort Analysis	To be completed by the patient in order to provide his/her thoughts and feelings with regard to several issues related to comfort (emotions, anxieties & harm caused)
6	Interview and complementary questions	Includes both some complementary questions that are related to allergic reactions, excess heat & sweat caused by the device Enables the user to provide his/her own suggestions on how the system being improved

**Table 5.** Pain-discomfort results using Borg-CR10 scale.

**Body Segment**	**Average**	**Body Segment**	**Average**	**Body Segment**	**Average**
1 Head	0	9 Lower Back	0.17	17 Left wrist	0.07
2 Face	0	10 Right Shoulder	0	18 Right Upper Leg	0
3 Neck	0	11 Left Shoulder	0	19 Left Upper Leg	0
4 Chest	0	12 Right Upper Arm	0	20 Right Lower Leg	0
5 Mid Torso-Waist	0.29	13 Left Upper Arm	0	21 Left Lower Leg	0
6 Lower Torso	0	14 Right Lower Arm	0	22 Right ankle	0.04
7 Upper Back	0	15 Left Lower Arm	0	23 Left ankle	0.06
8 Mid Back	0.03	16 Right Wrist	0.09	24 Overall (of the affected areas)	0.11

**Table 6.** Complementary questions asked to the patient during the informal interview.

**Problem Encountered**	**(No)**	**(Yes)**	**If Yes, Any Comments?**
User's allergy to the materials of the device	32	0	-
User's allergy to the materials used for the attachment	32	0	-
Materials caused sweating	32	0	-
Materials did not let the body “breath”	32	0	-
Materials absorbed water and took a long time to dry	32	0	
The attachment of the device was too tight	24	12	-Straps were too short
The device generated additional heat leading to excess sweating	32	0	-

**Table 7.** Overall results.

	**Metric**	**Units**	**Average**	**Level of Effect**
Energy cost	Perceived Exertion	Borg CR-10 score	0.71	Low

Biomechanical	Posture	REBA score	0	Low
	Localized pain/discomfort	Borg CR-10 score	0.11	Low

Comfort	Emotion	Average CRS score	1.4	Low
	Attachment	Average CRS score	2.5	Low
	Harm	Average CRS score	0.15	Low
	Perceived change	Average CRS score	0.67	Low
	Movement	Average CRS score	0.59	Low
	Anxiety	Average CRS score	1.63	Low

## References

[1] Tsipouras M.G., Tzallas A.T., Rigas G., Bougia P., Fotiadis D.I., Konitsiotis S. (2010). Automated Levodopa-induced dyskinesia assessment. Conf. Proc. IEEE Eng. Med. Biol. Soc..

[2] Pastorino M., Cancela J., Arredondo M.T., Pansera M., Pastor-Sanz L., Villagra F., Pastor M.A., Martin J.A. (2011). Assessment of bradykinesia in Parkinson's disease patients through a multi-parametric system. Conf. Proc. IEEE Eng. Med. Biol. Soc..

[3] Rigas G., Tzallas A.T., Tsipouras M.G., Bougia P., Tripoliti E.E., Baga D., Fotiadis D.I., Tsouli S.G., Konitsiotis S. (2012). Assessment of Tremor Activity in the Parkinson's Disease Using a Set of Wearable Sensors. IEEE Trans. Inf. Technol. Biomed..

[4] Cancela J., Pastorino M., Arredondo M.T., Pansera M., Pastor-Sanz L., Villagra F., Pastor M.A., Gonzalez A.P. (2011). Gait assessment in Parkinson's disease patients through a network of wearable accelerometers in unsupervised environments. Conf. Proc. IEEE Eng. Med. Biol. Soc..

[5] World Health Organization The Global Burden of Disease 2004. http://www.who.int/healthinfo/global_burden_disease/2004_report_update/en/.

[6] WHO Atlas: Country Resources for Neurological Disorders 2004. http://www.who.int/mental_health/neurology/epidemiology/en/.

[7] Polisena J., Coyle D., Coyle K., McGill S. (2009). Home telehealth for chronic disease management: A systematic review and an analysis of economic evaluations. Int. J. Technol. Assess. Health Care.

[8] Tugwell C. (2008). Parkinson's Disease in Focus.

[9] Cancela J., Pastorino M., Arredondo M.T., Nikita K.S., Villagra F., Pastor M.A. (2014). Feasibility Study of a Wearable System Based on a Wireless Body Area Network for Gait Assessment in Parkinson's Disease Patients. Sensors.

[10] Gan-Or Z., Giladi N., Rozovski U., Shifrin C., Rosner S., Gurevich T., Bar-Shira A., Orr-Urtreger A. (2008). Genotype-phenotype correlations between GBA mutations and Parkinson disease risk and onset. Neurology.

[11] Orr-Urtreger A., Shifrin C., Rozovski U., Rosner S., Bercovich D., Gurevich T., Yagev-More H., Bar-Shira A., Giladi N. (2007). The LRRK2 G2019S mutation in Ashkenazi Jews with Parkinson disease: is there a gender effect?. Neurology.

[12] Kandinov B., Giladi N., Korczyn A.D. (2009). Smoking and Tea Consumption Delay Onset of Parkinson's Disease. Park. Relat Disord.

[13] Shastry B.S. (2001). Parkinson disease: Etiology, pathogenesis and future of gene therapy. Neurosci. Res..

[14] Wider C., Wszolek Z.K. (2008). Etiology and pathophysiology of frontotemporal dementia, Parkinson disease and Alzheimer disease: Lessons from genetic studies. Neurodegener. Dis..

[15] Tsipouras M.G., Tzallas A.T., Karvounis E.C., Tsalikakis D.G., Cancela J., Pastorino M., Waldmeyer M.T.A., Konitsiotis S., Fotiadis D.I. A wearable system for long-term ubiquitous monitoring of common motor symptoms in patients with Parkinson's disease.

[16] PERFORM Project Website. http://www.perform-project.eu/.

[17] Goetz C., Poewe W., Rascol O., Sampaio C. (2003). The Unified Parkinson's Disease Rating Scale (UPDRS): Status and Recommendations. Society.

[18] Knight J.F., Deen-Williams D., Arvanitis T.N., Baber C., Sotiriou S., Anastopoulou S., Gargalakos M. Assessing the wearability of wearable computers.

[19] Gemperle F., Kasabach C., Stivoric J., Bauer M., Martin R. Design for Wearability. http://monet.cs.columbia.edu/courses/mobwear/resources/gemperle-iswc98.pdf.

[20] Baber C., Wilson J.R., Corlett E.N. (2005). Evaluation in Human-Computer Interaction.

[21] Borg G. (1990). Psychophysical scaling with applications in physical work and the perception of exertion. Scand. J. Work Environ. Heal..

[22] Hignett S., McAtamney L. Rapid Entire Body Assessment (REBA). http://www.ncbi.nlm.nih.gov/pubmed/10711982.

[23] Knight J.F., Baber C. (2005). A tool to assess the comfort of wearable computers. Hum. Factors J. Hum. Factors Ergon. Soc..

[24] Fransson-Hall C., Gloria R., Kilbom A., Winkel J., Karlqvist L., Wiktorin C. (1995). A portable ergonomic observation method (PEO) for computerized on-line recording of postures and manual handling. Appl. Ergon..

[25] Borg G. (1976). Simple rating methods for estimation of perceived exertion. Phys. Work Effort.

[26] Knight J.F., Williams D.D., Arvanitis T.N., Chris B., Wichmann A., Wittkaemper M., Herbst I., Sotiriou S. Wearability assessment of a mobile augmented reality system.

[27] De Bleser L., Vincke B., Dobbels F., Happ M.B., Maes B., Vanhaecke J., De Geest S. (2010). A New Electronic Monitoring Device to Measure Medication Adherence: Usability of the Helping Hand (TM). Sensors.

[28] Tharion W.J., Buller M.J., Potter A.W., Karis A.J., Goetz V., Hoyt R.W. (2013). Acceptability and usability of an ambulatory health monitoring system for use by military personnel. IIE Trans. Occup. Ergon. Hum. Factors.

[29] Abbate S., Avvenuti M., Light J. (2014). Usability Study of a Wireless Monitoring System among Alzheimer's Disease Elderly Population. Int. J. Telemed. Appl..

